# Smart Petri Nets Temperature Control Framework for Reducing Building Energy Consumption

**DOI:** 10.3390/s19112441

**Published:** 2019-05-28

**Authors:** Kheir Eddine Bouazza, Wael Deabes

**Affiliations:** 1 Department of Computer Science in Jamoum University College, Umm Al-Qura University, Makkah 25371, Saudi Arabia; wadeabes@uqu.edu.sa; 2 Laboratoire d’Informatique et des Technologies de l’Information d’Oran (LITIO), University of Oran, Oran 31000, Algeria; 3 Computers and Systems Engineering Department, Mansoura University, Mansoura 35516, Egypt

**Keywords:** petri nets, PID, air conditioning, energy consumption, smart systems

## Abstract

Energy consumption is steadily increasing in the Kingdom of Saudi Arabia (KSA), which imposes continuous strains on the electrical load. Furthermore, consumption rationalization measures do not seem to improve the situation in any way. Therefore, the implementation of energy saving policies become an urgent need. This paper targets developing a smart energy-saving framework for integrating new advanced technologies and conventional Air Conditioning (AC) systems to achieve a comfortable environment, optimum energy efficiency and profitability. In this paper, a three-stage smart control framework, which allows controlling room temperature according to the user’s preferences, is implemented. The first stage is a user identification process. In the second stage, a Petri Nets (PN) model monitors users and sends their preferred temperatures to the third stage. A PID controller is implemented in the third stage to regulate room temperatures. The interconnected sensing and actuating devices in this smart environment are configured to provide users with comfort and energy saving functionality. Experimental results show the good performances and features of the proposed approach. The proposed smart framework reduces the energy consumption of the current ON/OFF controller (219.09 kW) by a significant amount which reaches (116.58 kW) by ratio about 46.79%. Reducing energy consumption is one of these important features in addition to system reactivity and user comfort.

## 1. Introduction

The rapid growth in energy consumption in KSA puts continuous strains on the electrical load. This will force to double the production capacity every ten years if the status persists [1]. Furthermore, consumption rationalization measures do not seem to improve the situation in any way. Therefore, the implementation of energy conservation policies becomes an urgent need. Indeed, as the population grows annually on average by 2.1%, so does the total consumption of residential subscribers, from 42,820 kWh in 2009 to 47,563 kWh in 2014 [2].

**Figure 1:**
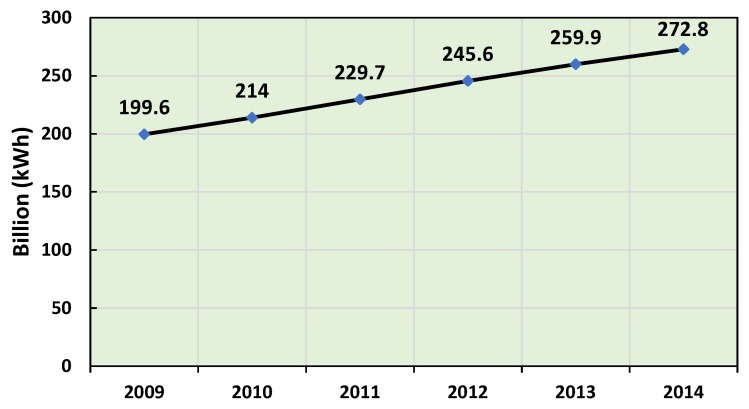
KSA Electricity Consumption [2].

The climate in the Kingdom of Saudi Arabia is a desert climate where there is an extreme heat throughout the day; the heat rises very quickly at sunrise and remains so until sunset. In summer, the average temperature is around 45 ${}^{\circ }$C, but the ambient air temperature can reach 50 ${}^{\circ }$C [1]. As a result, energy consumption in Saudi Arabia is steadily increasing. Figure 1 shows that global consumption is expected to increase with an average annual growth of 6.4%, from 199.6 billion kWh in 2009 to about 272.8 billion kWh in 2014 [2].

The western region of the kingdom has the highest electricity consumption level with about 38% of the total consumption [2]. Electricity is an energy source for 99% of end-users, of which 80% consume less than 4000 kWh per month and 1.4% consuming more than 10,000 kWh per month.

The residential sector consumes most of the public energy. Indeed, it is well known that the major part of energy consumption in homes is due to the use of heating/cooling of space and water, to the use of household appliances and lighting [3]. The city of Makkah, the largest city in the western region, had seen its electricity consumption increases at a rate of 12%. Also, the residential sector consumes more than 50% of total electricity production in the Kingdom. In summer, air conditioning systems are responsible for consuming 60–70 percent of the total energy consumed by residential buildings [1]. As a result, the Kingdom’s electricity sector must respond effectively to the regularly growing demand of electricity.

A smart environment is considered as an intelligent agent that recognizes behavior of occupants and a state of physical surroundings using sensors [4]. After that, the environment is adapted using controllers to optimize a specific measured performance. Recently, researchers were able to design smart environment test beds that track a location and activities of occupants and respond to hazardous situations. Diverse types of sensors are deployed in these test beds to classify different types of activities [5]. Usually, attached sensors to the occupant’s body are used to recognize the repetitive body activities, such as walking and running. However, data collected from sensors attached to objects (doors, windows, appliances, and medicine containers) surrounding the occupant are applied to differentiate other activities. Today, the rapid growth of processors and network communication technologies has had a significant impact on the configuration of intelligent environments [6]. Therefore, the three main factors that have affected the recent development of intelligent environments are, first, the availability of small, inexpensive, and easy-to-install network devices. Second, home accessibility of several networks technologies (Wi-Fi, Bluetooth, and Ethernet on-line), and, third, the dissemination of small computing devices such as smartphones, tablets, and netbooks.

An intelligent controller based on a Random Neural Network (RNN) on an Internet-of-Things (IoT) platform integrated with cloud processing for the formation of the RNN was presented in [7]. This IoT platform was integrated with cloud processing for RNN training. The proposed platform consumes 27.12% less energy than simple rule-based controllers. In [8], the authors tried to give new modeling of the smart home, to obtain greater flexibility to the user, which results in finer control of movable devices and thermostatic charge temperatures. A power management controller to optimize power consumption and demand-side management was presented in [9]. Reduction of energy consumption, and the cost of lighting in HVAC systems have been achieved using fuzzy logic. Authors in [10] have synthesized the relevant emerging themes of smart home technologies in crucial areas of users’ lives. In a critical and reasonably comprehensive way, the review of the functions, services, smart home was done, as well as the presentation of the benefits and the implementation.

Petri Nets (PN) are a promising tool widely used in the supervision and control of the discrete event control systems because of their great ability to represent and model concurrent and parallel processes. Authors in [11] presented a survey on the techniques used in the forcing event approach based on Ladder diagram, PN, and Colored PN (CPN). In [12] a real-time PN was used to provide a direct plant interface. An ordered CPN based controllers were also used to provide a direct plant interface in [13]. The modeling and analysis of manufacturing systems are one of the areas that have made extensive use of PN [14]. Indeed, PNs have been used to model production systems with buffers, automated assembly chains and for performance analysis of competing systems [15]. The formalism has undergone several extensions to meet two objectives. First, model, control and supervise complex systems describe many areas of application. Second, to deal with the emergence of new systems such as real-time systems. Among these extensions are the Stochastic PN (SPN) [16]; the CPN [17]; the Temporal PN (TPN) [18], etc.

As for the smart homes, PNs have not been widely used as the previously mentioned research areas. However, in [19], PN models for the most used electrical appliances were considered. Indeed, the author was able to show the working principles and the interactions between the refrigerator, the washing machine, and the smart home system. Using the PN model, a real-time Energy Management Systems (EMS) that reduces the cost of electrical energy, through prioritization of resources and simultaneous interference with smart electrical appliances, is presented in [20]. Integrating the qualities and features of smart home appliances and the PN, an appliance-based home energy management system is proposed in [21]. In order to model and analyze discrete event control systems in smart homes, the authors used PN in [22]. In [23], a synchronized PN for modeling a smart conference scenario was used. In [24], using the colored hybrid PN, the different processing and signal state changes in a hybrid inverter HVAC (Heating, Ventilation, and Air Conditioning) system were modeled. The discrete and continuous modes of the model have been executed together. In [25], using the PN, the authors modeled HVAC control systems, in order to analyze the performances of the control system and thus were able to find the optimal temperature for minimal energy consumption. It is well known that inadequate control loop can lead to wasted energy and reduce the quality of user comfort [26].

The Predictive Control Model (MPC) is one of the most used methods for controlling HVAC systems. It works on adjusting the controller’s response according to the prediction of the future behavior of the building [27,28,29]. However, despite the good performance of the MPC, it suffers from some disadvantages. One of the most important is the sensitivity of this control method to the modeling of uncertainties that can not be ignored [30]. The importance of the model accuracy comes from the fact that a mismatch in the model will imply an inaccurate prediction and therefore deterioration of the performance of the MPC. Another major problem of this control method is a large number of calculations necessary for its implementation, which requires a shift to local solutions such as distributed MPC [30]. Indeed, when the complexity of the building model become important, the lack of computing resources in building control platforms makes it impossible to calculate the centralized MPC. One way to overcome this problem is to use numerical solvers associated with specific hardware which increases the cost of this solution. Another way to solve this second problem is to use local solutions such as distributed MPC. However, these local solutions may be less accurate than the global method [31,32].

In this paper, to reduce energy consumption, a framework for smart temperature control is developed. This framework is based on the use of three different stages. The first stage is used to recognize the user and then generate his preferred temperature. In the second stage, a PN uses the information collected from the first stage generates the desired temperature profiles of the users and sends it as a reference signal to the next stage. The third stage is a regulation stage using a PID controller works on matching the actual room temperature, and the generated user’s preferred temperature.

After recalling some PN, PID controller and face recognition properties in Section 2, Section 3 and Section 4, the three stages of the framework are theoretically and practically presented and explained each in separate sections. Numerical results explained in Section 7 will show the good performances achieved by applying the proposed framework. The final section will conclude this work and provide some directions for future works.

## 2. Petri Nets

**Figure 2:**
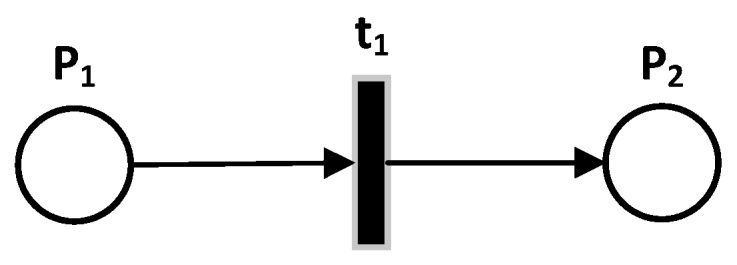
Petri Net.

A PN is a directional bipartite graph consisting of a finite set of places, $P=P_{1},P_{2},P_{3},\ldots ,P_{m}$, represented by circles, a finite set of transitions, $T=t_{1},t_{2},t_{3},\ldots ,t_{n}$, represented by bars, (P∩T=∅ and P∪T=∅), and a finite set of oriented arcs that links a place to a transition or a transition to a place. These arcs represent the conditions required for an action to be feasible and the consequences of this action, as shown in Figure 2.

**Figure 3:**
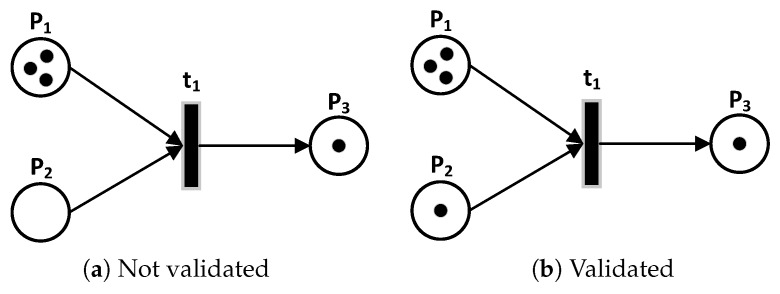
Transitions Validation.

**Figure 4:**
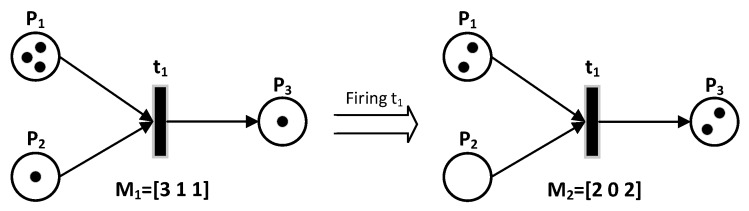
Firing a Transition.

*N*:*PT* ⟶ χ is the input matrix that specifies the arcs directed from places to transitions, O:PT⟶χ is the output matrix that specifies the arcs directed from transitions to places. Here, χ is the set of non-negative integer numbers. A condition is the description of the state of a modeled system resource. In the PN formalism, the condition is modeled with the help of a place. An event is an action that takes place within the system and whose realization depends on the state of the system. In the PN formalism, the event is modeled using a transition. The marking of a PN is specified by the presence within the places of a finite number (positive or zero), marks, or tokens. A place is empty or marked. During the system evolution, the marking is likely to be modified. The initial marking, $M_{0}$, of a PN corresponds to the initial distribution of the tokens in each of the PN places, which specifies the initial state of the system. The evolution of the PN state corresponds to the evolution of the marking. Tokens, which materialize the state of the network at a given moment, can pass from one place to another by crossing or firing a transition, as shown in Figure 3. A transition is validated (or fired) if all its entry places contain at least one token. Passing or firing a transition involves removing one token from each of the transition’s input places and adding one in each of the same transition’s output places, as shown in Figure 4.

## 3. Pid Controller

When process control appeared around the 1940s, the PID controller immediately became one of the most used controllers. Indeed, currently, in all applications requiring control, more than 95% of the used controllers are of PID type [33]. PID controllers have been able to adapt to many technological changes over time. They have been applied to many fields such as power generation, mechanics, transportation, pneumatics, crushing processes, microprocessors, electronic tubes, integrated circuits as well as in other application areas (for more details see [34,35,36]).

Almost all PID controllers manufactured currently are fact-based microprocessors which have opened up new opportunities and allowed the use of additional features such as continuous adaptation planning gain and automatic tuning [37,38,39,40]. There have been some publications on expanding the idea of crushing process control from the crusher itself, such as in [34,41].

The PID is described by the following equation [42]:

(1) $$u\left(t\right)=k_{p}e\left(t\right)+k_{i}\int_{0}^{t}e\left(\tau \right)d\tau +k_{d}\frac{de(t)}{d_{t}}$$

where *t* is the time or instantaneous time (the present), τ is the variable of integration (takes on values from time 0 to the present *t*). u(t) is the control signal, e(t) is the control error (e(t)=r(t)−y(t)) when r(t) is the reference variable, and y(t) is the system measurement variable, and $k_{p}$, $k_{i}$, and $k_{d}$ are all non-negative and they denote the coefficients for the proportional, integral, and derivative terms respectively (sometimes denoted P, I, and D). The system control signal is the sum of these three terms: the P-term (which is proportional to the error), the I-term (which is proportional to the integral of the error), and the D-term (which is proportional to the derivative of the error). The integral, proportional, and derivative part can be interpreted as control actions based on the past, the present and the future, respectively. Each term has a particular task:

- The P term ($k_{p}e\left(t\right)$) is proportional to the current value of the error e(t). For example, if the error is large and positive, the control output will be proportionately large and positive, taking into account the gain factor “$k_{p}$”. Using proportional control alone will result in an error between r(t) signal and y(t) because it requires an error to generate the proportional response. If there is no error, there is no corrective response.
- The I term ($k_{i}\int_{0}^{t}e\left(\tau \right)d\tau$) accounts for past values of the error and integrates them over time. For example, if there is a residual error after the application of proportional control, the integral term seeks to eliminate the residual error by adding a control effect due to the cumulative historical value of the error. When the error is eliminated, the integral term will cease to grow. This will result in the proportional effect diminishing as the error decreases, but this is compensated for by the growing integral effect.
- The D term ($k_{d}\frac{de(t)}{d_{t}}$) predicts system behavior and thus improves settling time and stability of the system. It is also the best estimate of the future trend of the error, based on its current rate of change. It is sometimes called “anticipatory control”, as it is effectively seeking to reduce the effect of the error by exerting a control influence generated by the rate of error change. The more rapid the change, the greater the controlling or dampening effect.

## 4. User Identification

The automatic management of user access to restricted systems can be divided into two broad categories: authentication and recognition/identification. In authentication systems, the system must give a binary decision whether or not the subject belongs to a group of authenticated users. It is not necessary to identify exactly who the subject is. On the other hand, identification systems impose the exact determination of the subject identity.

User identification and inherently authentication can be done in mainly three ways: knowledge-based, token, and biometric [43]. Knowledge-based identification means the exploitation of “something that only the user knows”, such as a password or a personal identification number. The idea of token identification is based on the use of a small electronic device that identifies the person who wears it. Biometric data can be targeted specifically towards authentication, e.g., [44]; or identification, e.g., [45]. The advantage of this type of data is that it can not be intercepted or lost. The idea is actually to exploit data about “something that is only this user”, such as his voice, fingerprints or his images, especially those of the face. In the latter case, the required operations are acquisition, processing, and interpretation of these images.

For the proposed framework, the method used in stage 1, an identification method based on facial recognition is the best, as it does not require any special cooperation from the user. Indeed, facial recognition has been a very active area of research in the last four decades since the first system developed by Kanade [46]. These years of research have made it possible to reach an excellent level of maturity and a lot of success in several real-time practical applications [47,48].

## 5. Smart Temperature Control Framework

**Figure 5:**
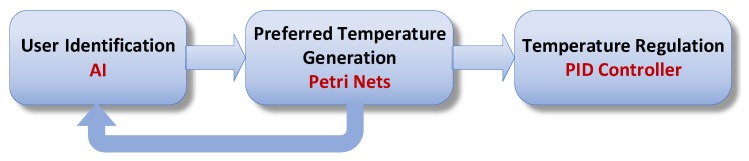
Temperature control framework.

In this section, the theoretical aspects of the framework are explained. More practical details will be given in the case study. The proposed framework consists of three stages, as shown in Figure 5, each dedicated to a specific mission.

The role of the first step is to recognize the user. Indeed, during this stage, an identification process allows to identify the detected person, and then to transmit the identification results to the next stage.

In the second stage, based on user identification results obtained from the previous stage, the user preferred temperature is generated and sent to the next step.

So, the main role of this stage is to monitor the room, to obtain the identification results, and based on these results, to send the identified user preferred temperature to the next step.

We chose, in this stage, to use the PN, for its qualities mentioned in Section 2, such as power of modeling and implementation, the possibility of real-time implementation and supervision qualities. Thus, thanks to the use of PN, we can while supervising and monitoring the room, send the preferred temperatures.

The third stage is the temperature regulation stage. In this paper, because of its simplicity and speed of convergence, as explained in Section 3, a traditional PID has been chosen. The latter will receive the user’s preferred temperature as a reference signal and send the control signals needed to regulate the temperature in the room.

Thus, we obtain a smart system, able, autonomously, to monitor the room, to detect a new presence, to identify the exposed person, to generate his preferred temperature, to send this temperature as a reference signal for temperature regulation and finally to make sure that the ambient temperature in the room reaches this reference temperature. Thus, we obtain a smart framework with various components as a single intelligent entity. This framework is autonomously able to:

- monitor the presence at the office,
- detect a new presence,
- identify the detected person,
- generate his preferred temperature,
- send this temperature as a reference signal for temperature regulation,
- guarantee that the ambient temperature in the room reaches this reference temperature,
- finally, goes into standby state or in the off state in the absence of anyone.

The innovation of this work is to have associated different separated processes; person identification, monitoring and supervising of the office through PN, and temperature regulation using PID controller to obtain a smart framework that reduces the energy consumption. All tools used in this framework are well-known and have given good results each in its field. We chose each of these components for its main advantages. Therefore, the proposed framework integrates all the benefits of these components: feasibility, ease of implementation, and high flexibility.

## 6. Case Study

**Figure 6:**
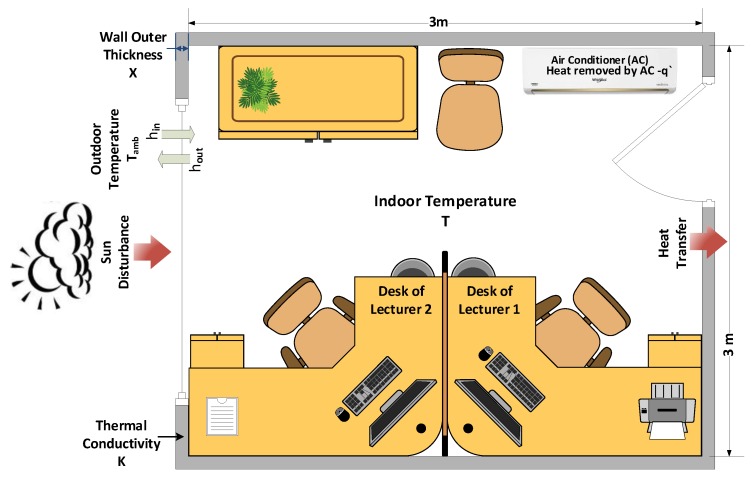
Office Layout.

As a case study, we consider our office located on the second floor of Al-Jamoum University college building with an area of 9 m${}^{2}$. The office, shown in Figure 6, is assigned to two lecturers Lec1 and Lec2. The office includes two desks, a window, a door, an Air Conditioner (AC), a PID controller connected to the AC, and sensors to measure the necessary data.

### 6.1. Stage 1: User Identification

The main idea of face recognition is to compare a face captured image with a collection of previously stored faces (a faces database) to identify the person in the input image (Al-Allaf, 2014). A standard PC camera, placed in front of each lecturer, is used to implement the automatic face identification system in real time. The resources needed to develop these applications can be retrieved from available open sources.

**Figure 7:**
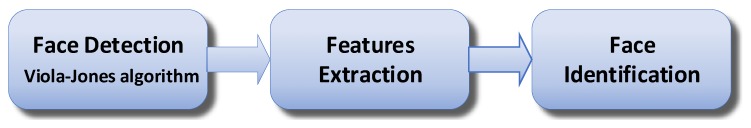
Identification steps.

The implementation is done according to the three steps shown in Figure 7:

Step 1: Face detection (using Viola-Jones algorithm)
Step 2: Feature extraction
Step 3: Face identification

### 6.2. Stage 2: Desired Temperature Generation Using a Supervisor PN

**Figure 8:**
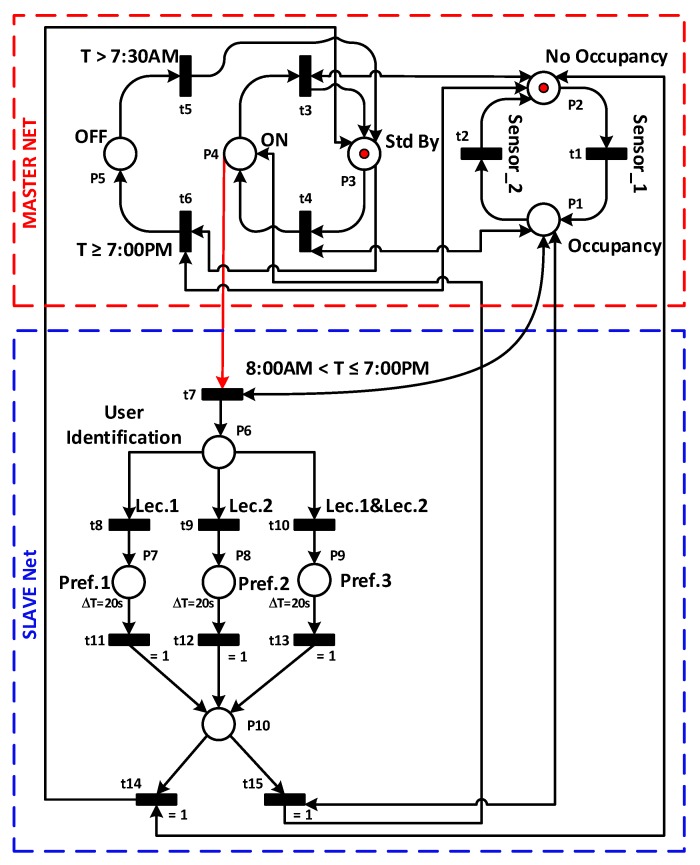
PN based Supervisor.

For simplicity, the supervisor PN shown in Figure 8 is divided into master and slave nets. The master net is represented by a red box, while the slave one is in a blue box. In Table 1, places and transitions of the supervisor PN-based are given.

#### 6.2.1. Master PN

The master net is designed to identify states of the system and to control the operating modes used for each different user’s presence situation. It is composed of five states. The Off state orders the extinction of the whole system at 7:00 p.m. while the system restarts at 07:30 a.m. From this time on, the system is either in ON state, enabled when the system detects an office occupancy, or in Standby state, activated when the system detects no occupancy.

The advantage of using the standby state is that the desired reference temperature (sent to the PID in stage 3) remains relatively close to the user’s preferred temperature. Thus, the proposed system has significant energy savings, especially when the state of non-occupancy lasts too long, compared to the On/Off mode that maintains the same reference temperature even without occupancy. At the same time keeping the system in standby state allows obtaining a reactive system that can reach the user’s preferred temperature swiftly after detecting a new presence in the office. If the system shuts down and hence stops sending a reference temperature when there is no one in the office and if this non-occupancy state is extended, then the office’s temperature will increase severely under the effect of the outdoor temperature (very high in this geographical region). As a result, the quality of the user’s comfort will decrease, in case any lecture has to come back to the office again since the system will take a long time to regulate the temperature. Besides, after the detection of a new presence, the return to the user preferred temperature will require too much time.

Consequently, the use of the standby state allows reaching the user’s preferred temperature quickly, which significantly improves the users’ comfort. A reference temperature equal to 30 ${}^{\circ }$C is sent during this state. The system is switched on at 7:30 a.m., and it goes directly into standby state which prepares the office for the arrival of the lecturers around 8:00 a.m.

#### 6.2.2. Slave PN

The slave networks only when the master net goes into the ON state and a fast user identification process starts. At the end of this process, the framework will finish identifying the lecturer’s presence in the office.

There are three exclusive cases:

- The first case is that the identified lecturer is Lec1, then his preferred reference temperature T1 will be sent to the third stage (PID controller).
- The second case when Lec2 is identified in the office. In this case, the preferred reference temperature sent to the third stage will be T2.
- In the last case the Lec1 and Lec2 will be present in the office simultaneously, then the reference temperature T3, on which Lec1 and Lec2 have agreed, will be sent to the third stage.

The sending state of the reference temperature to the third stage will remain activated for a duration chosen by the system manager (in our case a duration equal to 20 s). At the end of this period, two situations are possible. The first one is that a presence remains detected in the office, a new identification step is then performed, followed by sending the preferred reference temperature of the identified user and so on. The second possible situation is that no presence is detected in the office, then standby mode will be activated.

### 6.3. Stage 3: Temperature Regulation Based on a PID Controller

The open loop transfer function of an air conditioner can be represented as [49]:

(2) $$TF_{1}=\frac{1}{M.C.s+m^{{}^{\prime }}C_{p}+\frac{KA}{X}}$$

where *M* is the thermal mass (including the weight of the users and the thermal mass of indoor air), *C* is the total specific heat capacity, *A* is the indoor surface area, *X* is the outdoor wall thickness, *K* is the thermal conductivity coefficient, $m^{{}^{\prime }}$ is the exchanging air flow and $C_{P}$ heat capacity at constant pressure *p* used to deduce the enthalpy of the gas (thermodynamics).

**Figure 9:**
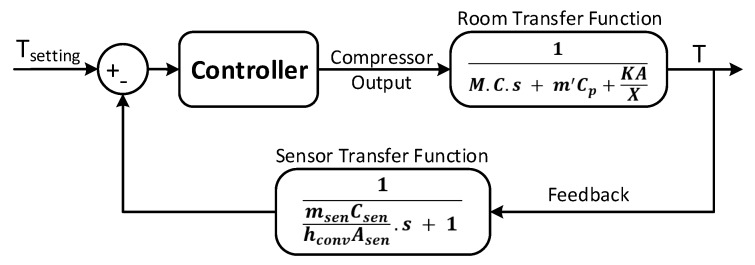
AC closed loop system.

To obtain the closed-loop model, a sensor in the evaporator of the indoor unit of a split type air conditioner is installed. The sensor transfer function is as follow:

(3) $$TF_{2}=\frac{1}{\frac{m_{sen}C_{sen}}{h_{conv}A_{sen}}.s+1}$$

where $m_{sen}$ is the sensor mass, $C_{sen}$ is the sensor heat capacity, $h_{conv}$ is the convection coefficient and $A_{sen}$ is the sensor area. Therefore, the obtained closed loop system, necessary for the office temperature regulation, is shown in Figure 9.

**Figure 10:**
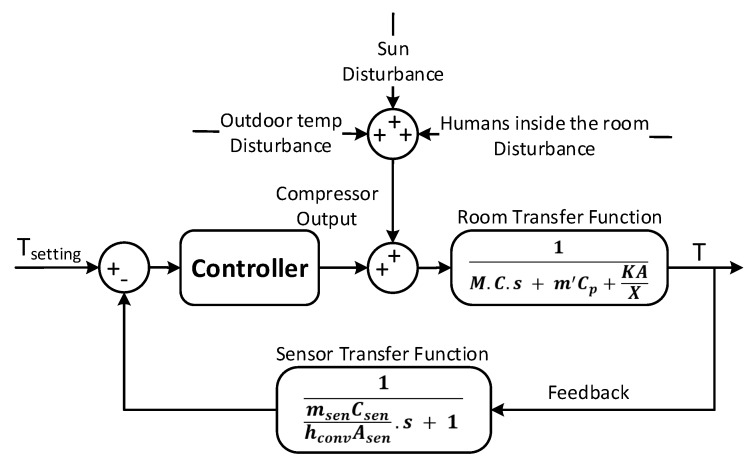
AC closed loop system with Disturbances.

In addition, a heat loss could occur through walls and windows. Therefore, to get as close to reality as possible, three different types of disturbances are added to the previous model, as shown in Figure 10. Indeed, the influence of the outside temperature $D_{Out}$, the heat released by the human $D_{Hum}$ and the solar radiation passing through the window $D_{Sun}$ are the disturbances that have been added over the control signal.

Typically, the outside temperature affects the office’s temperature as one of the most common disturbances in such type of systems. It is well known from the zero laws of thermodynamics that the heat goes from warm to cold. In Saudi Arabia, the average temperature per year is about 41.6 ${}^{\circ }$C which is very high compared to the general temperature inside a typical office, therefore there will be a heat-transfer into the office during a long time of the year.

Regarding the human disturbance inside the office, in order to maintain the human’s functionality, their bodies’ temperature has to be kept at a constant level. Therefore, thermal radiations from people affect the internal temperature of the office. Typically, a person releases about 70 W during sleeping and 100 W during doing light effort, which is considered in this work.

Another type of disturbance that can affect temperature control is the solar radiation. There is a direct effect for the sun heat which increases the office temperature. This disturbance can be modeled from the solar constant on the planet’s surface which is around 684 W/m${}^{2}$. Taking into consideration the effect of the clouds and windows reflection, about 40% of the total incoming energy during will enter the office.

## 7. Simulation Results

In this section, simulations results are presented. These results demonstrate the effectiveness of the smart temperature control framework which reduces the energy consumption while respecting the user’s preferences. For these simulations, Matlab 2018a and PIPE: Platform Independent Petri Net (v4.3.0) is used to implement the system.

The office presented in Figure 6 is supervised using the PN model developed in Figure 8. The regulation model applied in stage 3 of the framework is represented by the model developed in [49] where a Hitachi RAS-22SCT/RAC-22SCT AC was used. This model is characterized by high quality, precision, and fits precisely with our needs. Indeed, the area of the room is similar to the one used in [49], with some changes in the weight and the number of people present in the office. For the biometric identification, an image of the present lecturer is captured by a webcam and the Viola-Jones algorithm is applied to detect the lecturer’s face. Afterward, the identification process is carried out to identify the corresponding lecturer and generate his preferred temperature.

**Figure 11:**
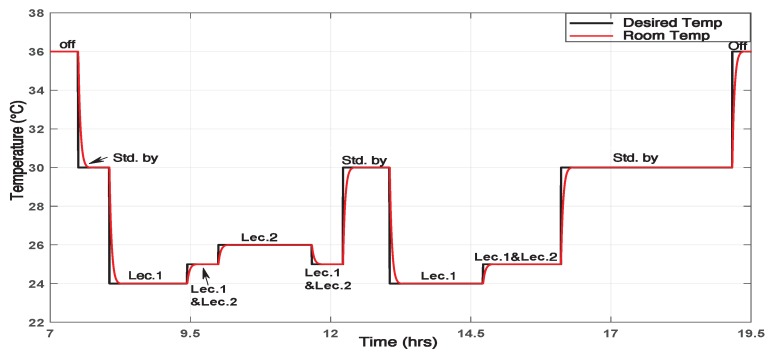
Room temperature response with PID controller.

The scenario of lecturers presence implemented in the simulations is that of a typical working day. The scenario is described in Figure 11.

Whenever a lecturer is detected, his preferred temperature is sent as a reference signal to stage 3. Lec1 has chosen 24 ${}^{\circ }$C as a preferred temperature while the lec2 has chosen 26 ${}^{\circ }$C. When both lecturers are present in the office simultaneously, they have chosen 25 ${}^{\circ }$C as their preferred temperature. The AC always starts working in Stand By (Stdby) mode at 7:30 a.m. to reduce the office’s temperature to be suitable for the incoming lecturers. Usually, the working day ends at 4:00 p.m., however, the AC stays at the Stdby mode to keep a moderate temperature in case a lecturer returns to the office. It is crucial to notice that in this case, immediately after detecting the lecturer presence, the framework sends his preferred temperature to stage 3). At 7:00 p.m. the day is over and the AC goes into OFF mode.

**Figure 12:**
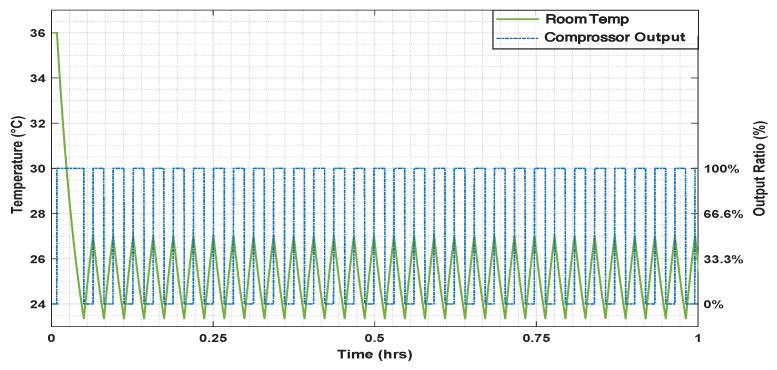
The room temperature response and compressor output with On-Off control.

Figure 12 shows the office’s temperature when the ON/OFF controller is applied. In Figure 11 the effect of the PID controller on the temperature according to the presence changes is shown.

**Figure 13:**
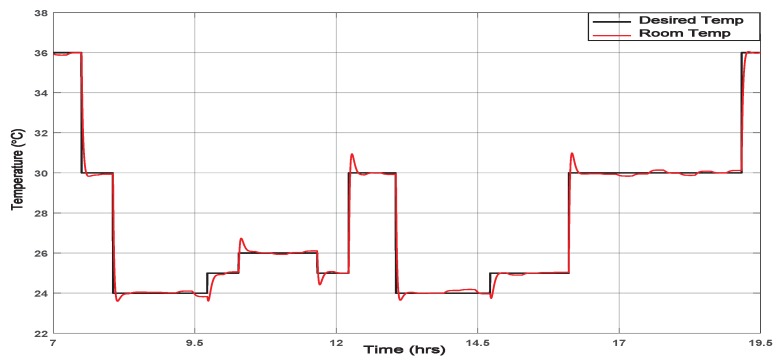
Effect of disturbances on the room temperature regulation.

The temperature simulation using the smart framework with the external disturbances is shown in Figure 13.

Figure 13 shows the effect on the output temperature due to the disturbances explained in Section 6.3. However, there is small overshoot in the temperature at transition points; the control signal can overcome that disturbance and enforce the output temperature to follow up the desired profile smoothly.

**Figure 14:**
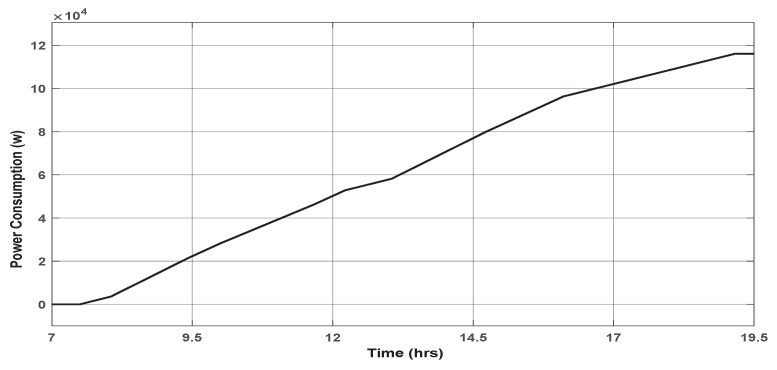
The power consumption after applying PID controller.

**Figure 15:**
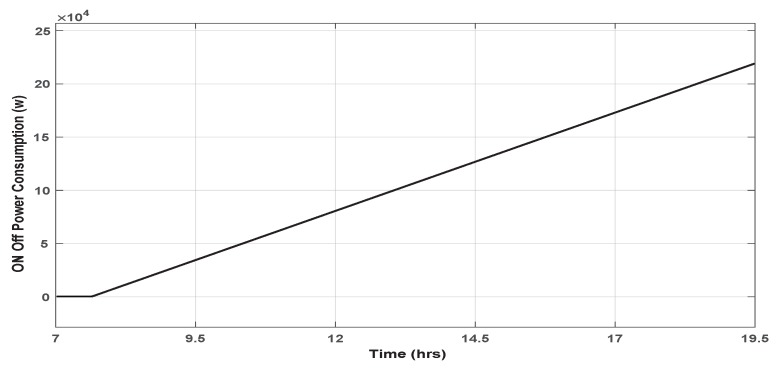
The power consumption of ON-OFF controller.

Figure 14 illustrates the energy consumed after applying the PID controller within the smart framework, while Figure 15 illustrates the energy consumed when the On/Off control was applied.

The simulation results prove that the main two objectives of the proposed framework; the user’s comfort and energy consumption reduction are achieved.

1. The user’s comfort can be observed in Figure 11 through the short response time in the temperature signal. Indeed, it can be seen that, at each change of lecturer presence in the office, the framework generates the new user’s desired temperature, and the PID controller acts on the office ambient temperature to reach the desired one quickly. Choosing a room temperature equal to 30 ${}^{\circ }$C in the absence of both lecturers (Stdby mode), participates in reaching the preferred user’s temperature quickly, since it is well known that the outside temperature in KSA can easily exceed 50 ${}^{\circ }$C during summer. This reasonable ambient temperature consumes more energy, but it is necessary to maintain an acceptable user comfort level.
2. Energy saving: The comparison between the traditional system based on an On/Off signal shown in Figure 15 and the proposed smart framework illustrated in Figure 14 shows a considerable reduction in the energy consumed by the latter compared to the first one. In fact, the simulations show that using the smart framework, the reduction in energy consumption is about 46.79% compared to the current On/Off controller. Indeed, using the On/Off controller, to keep the temperature around a predefined temperature of 25 ${}^{\circ }$C during 12 h, from 7:30 a.m. to 19:30 p.m., the total consumed energy was of 219.09 kW as shown in Figure 15, while it is only of 116.58 kW using the smart framework, as shown in Figure 14.

**Remark** **1.** *To ensure an acceptable office ambient temperature throughout the day, we have chosen, in the absence of the two lecturers, a temperature equal to 30 ${}^{\circ }$C. This choice allows reaching the two lecturers preferred temperatures as quickly as possible. Choosing a lower ambient temperature forces the system to use much airflow at the output of the AC, which dramatically affects the lecturers’ comfort.*

**Figure 16:**
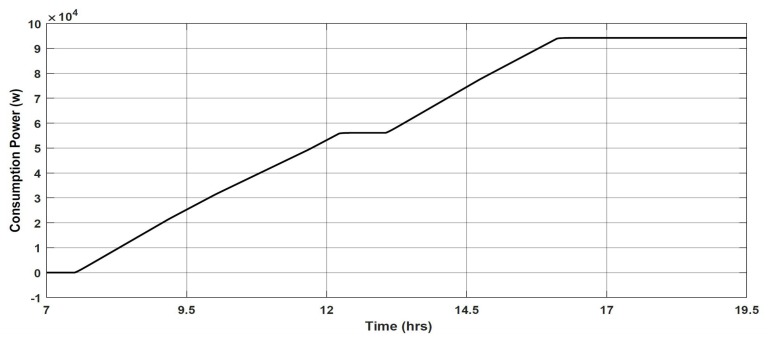
Power consumption after applying the PID controller at Amb, Temp = 36 ${}^{\circ }$C.

However, keeping the ambient temperature at 30 ${}^{\circ }$C costs much more power consumption since the temperature in our region regularly exceeds 50 ${}^{\circ }$C. Indeed, as shown in Figure 16, choosing an ambient temperature equal, for example, to 36 ${}^{\circ }$C allows, using the smart framework, to consume only 94.10 kW over the same duration, which represents a saving of 57.05% compared to the On/Off mode.

## 8. Framework Advantages

**Figure 17:**
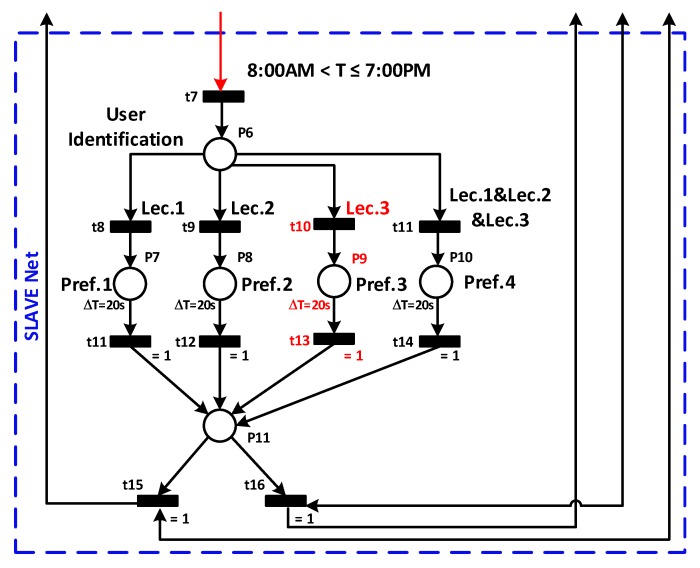
Modified PN to accommodate *Lec*3.

- **Energy consumption reduction:** Indeed, compared to the On/Off method used currently in the department building based on a predefined constant reference temperature throughout the day, the proposed framework allows sending each user preferred temperature. As a result, very significant energy savings are made, as shown in Figure 14. Beyond these savings, the most important is the idea that at any time of the day, the proposed smart framework consumes the exact required energy, neither more nor less.
- **User’s comfort:** The proposed approach responds precisely to the users’ needs. Indeed, the role of the second stage, the PN based supervisor, is to detect any presence in the office, identify the user, then send the corresponding reference temperature expressed by the identified user. This dramatically improves user comfort.
- **Reactivity:** One of the critical features of the proposed approach is its reactivity. Indeed, this approach has a great ability to react, very quickly, to changes in its environment. This is because through the use of a PN and a closed control loop, can simultaneously perform several functions of observation, monitoring, and correction of the system. Any changes in the presence or absence of people in the controlled space are immediately reflected in the reference temperature sent to the third stage as well as in the control signal sent to the controlled system.
- **System Supervision:** The fact that PN and closed-loop control are implemented in this framework gives the ability to supervise the global system in real time to ensure that the issued command is in perfect harmony with the state of the system. This avoids the propagation of component failure to other components, which provides an entirely reliable control system.
- **Flexibility:** The great interest of the proposed framework is its flexibility. Indeed, any necessary modification of conditions or variables used in the framework can be easily implemented in the PN and taken into account in office temperature regulation. Such modification could be for example the increasing or decreasing of the number of people using the office, or changing the preferred temperature of one or all users. In the case study discussed in this paper, if we decide to increase the number of people using the office to three, then we have to make the following changes. First, adding an additional state, with its input and output transitions, corresponding to the sending of the new user’s preferred temperature as a reference temperature (in a case he is alone in the office). Second, changing the preferred temperature on which the three users have agreed. We get, as a result, the following PN shown in Figure 17.

## 9. Conclusions

A smart framework that controls room temperature based on user preferences has been developed. The framework is composed of three stages. The first stage identifies the user and generates his preferred temperature. The second stage, based on the PN, is responsible for the supervision and monitoring of the controlled room and sends the users’ preferred temperatures as a reference signal to the next stage. The controller in the third stage regulates the temperature of the room. Simulation results prove the interest of the framework, its relevance, and convincing performances. Indeed, the results showed that the proposed smart framework, in addition to being fast, flexible and reactive, allowed to achieve significant energy savings. Another advantage of the proposed approach is that while achieving significant energy savings, it improves the users’ comfort by providing them with their exact preferred temperatures. In future work, we have two objectives. First, applying the proposed framework using the developed Petri Nets as well as more advanced regulation tools will undoubtedly improve performance. Typically, the improvement is due to the integration of more parameters from the controlled environment into the framework. Second, the proposed framework will be extended to control all the administrative offices of the Computer science department.

## Figures and Tables

**Table 1:** Developed PN places and transitions.

Places		Transitions	
$P_{1}$	An occupancy in the office	$t_{1}$	User detection
$P_{2}$	No occupancy in the office	$t_{2}$	No user detection
$P_{3}$	Stand-by mode	$t_{3}$	Working status changes from ON to Standby
$P_{4}$	ON mode	$t_{4}$	Working status changes from Standby to ON
$P_{5}$	OFF mode	$t_{5}$	Working status changes from OFF to Standby
$P_{6}$	User identif. process	$t_{6}$	Working status changes from Standby to OFF
$P_{7}$	Sending user1 desired temp. to stage3 during *t* = 10 min	$t_{7}$	Beginning of the identification
$P_{8}$	Sending user2 desired temp. to stage 3 *t* = 10 min	$t_{8}$	Lecturer 1 detected
$P_{9}$	Sending users 1 and 2 desired temp. to stage 3 *t* = 10 min	$t_{9}$	Lecturer 2 detected
$P_{10}$	Intermediary state	$t_{10}$	Lecturer 1 and 2 detected simultaneously
		$t_{11}$, $t_{12}$ and $t_{13}$	=1
		$t_{14}$ and $t_{15}$	=1

## Abbreviations

The following abbreviations are used in this manuscript:

AC	Air Conditioner
HVAC	Heating, Ventilation and Air Conditioning
RNN	Random Neural Network
IoT	Internet of Things
PN	Petri Nets
CPN	Colored Petri Nets
SPN	Stochastic Petri Nets
TPN	Temporal Petri Nets
MPC	Predictive Control Model
*Lec*1	Lecturer #1
*Lec*2	Lecturer #2
HVAC	Heating, Ventilation and Air Conditioning
PID	Proportional, Integral, and Differential Control
Stdby	Stand By mode

The following symbols are used in this manuscript:

*P*	Set of places in Petri Net
*T* (in PN)	Set of transitions in PN
*t*	is the time or instantaneous time (the present) (s)
τ	is the variable of integration (takes on values from time 0 to the present *t*) (s)
e(t)	Control error
r(t)	Reference signal
y(t)	System measurement variable
$k_{p}$	PID proportional constant
$k_{i}$	PID integral constant
$k_{d}$	PID derivative constant
$TF_{1}$	Transfer #1
$TF_{2}$	Transfer #2
*M*	Mass (kg)
*C*	Total specific heat capacity (J·kg${}^{-1}$·${}^{\circ }$C${}^{-1}$)
$C_{p}$	Heat capacity at constant pressure J/${}^{\circ }$C)
*T* (in TF)	Temperature (${}^{\circ }$C)
$m^{{}^{\prime }}$	Exchanging air flow (m${}^{3}$/s)
*A*	Indoor surface area (m${}^{2}$)
*X*	Outdoor wall thickness (m)
*K*	Thermal conductivity coefficient (W·m${}^{-1}$·${}^{\circ }$C${}^{-1}$)
$m_{sen}$	Sensor mass (kg)
$C_{sen}$	Sensor heat capacity (J/${}^{\circ }$C)
$h_{conv}$	Convection coefficient (W/(m${}^{2}$ ${}^{\circ }$C))
$A_{sen}$	Sensor area (m${}^{2}$)
